# Comparison of outcomes after anterior cervical discectomy and fusion with and without a cervical collar: a systematic review and meta-analysis

**DOI:** 10.1186/s13018-024-04661-8

**Published:** 2024-03-07

**Authors:** Tingxin Zhang, Gang Gao, Yanhong Li, Feng Gao, Wupeng Yang, Yongjiang Wang, Nana Guo

**Affiliations:** 1 Department of Orthopedics, Ordos Central Hospital, 23 Ekin Hollow West Street, Ordos, 017000 China; 2 Department of Critical Care Medicine, Ordos Central Hospital, 23 Ekin Hollow West Street, Ordos, 017000 China

**Keywords:** Cervical collar, Anterior cervical decompression and fusion, Meta-analysis, Systematic review

## Abstract

**Purpose:**

The clinical outcomes of patients who received a cervical collar after anterior cervical decompression and fusion were evaluated by comparison with those of patients who did not receive a cervical collar.

**Methods:**

All of the comparative studies published in the PubMed, Cochrane Library, Medline, Web of Science, and EMBASE databases as of 1 October 2023 were included. All outcomes were analysed using Review Manager 5.4.

**Results:**

Four studies with a total of 406 patients were included, and three of the studies were randomized controlled trials. Meta-analysis of the short-form 36 results revealed that wearing a cervical collar after anterior cervical decompression and fusion was more beneficial (*P* < 0.05). However, it is important to note that when considering the Neck Disability Index at the final follow-up visit, not wearing a cervical collar was found to be more advantageous. There were no statistically significant differences in postoperative cervical range of motion, fusion rate, or neck disability index at 6 weeks postoperatively (all *P* > 0.05) between the cervical collar group and the no cervical collar group.

**Conclusions:**

This systematic review and meta-analysis revealed no significant differences in the 6-week postoperative cervical range of motion, fusion rate, or neck disability index between the cervical collar group and the no cervical collar group. However, compared to patients who did not wear a cervical collar, patients who did wear a cervical collar had better scores on the short form 36. Interestingly, at the final follow-up visit, the neck disability index scores were better in the no cervical collar group than in the cervical collar group.

PROSPERO registration number: CRD42023466583.

## Introduction

Cervical degenerative disc disease (CDDD) is a prevalent spine condition that often necessitates surgical intervention for patients with severe symptoms [1]. Surgery is still considered the gold standard for patients who do not respond to conservative treatments or who experience severe myelopathy [2]. Anterior cervical decompression and fusion (ACDF) was initially introduced by Smith and Robinson [3] in 1957 at the 24th American Academy of Orthopaedic Surgeons (AAOS) conference. In June of that same year, Ralph Cloward reported the ACDF technique using SA derived from a fresh cadaver [19]. ACDF has since become the established and widely accepted surgical approach for treating CDDD [20, 21]. Although ACDF is a commonly performed spinal surgery and steel plates have biomechanical advantages, the use of cervical collars (CCs) after ACDF is still widely accepted [4, 5], but there is still no consensus on whether CCs should be used after surgery. The purpose of using CCs after ACDF is to prevent further spinal cord injury, promote vertebral body ossification, reduce pain, and ultimately provide patients with more stability [6, 7]. However, it is important to note that the routine use of postoperative CCs is still primarily based on routine practice and surgeon preference rather than on reliable evidence-based literature [8]. There is still insufficient level-one evidence to prove the proposed advantages of CCs after ACDF. Previous meta-analyses have compared the benefits and drawbacks of wearing a CC after ACDF surgery [22, 23]. However, all previously published meta-analyses have significant limitations, as they did not compare patient postoperative quality of life as an outcome. Postoperative assessment of quality of life is an important aspect of evaluating the efficacy of treatment for cervical spondylosis [24]. Therefore, to compare the clinical outcomes of patients after ADCF, we conducted a meta-analysis and reviewed previous studies. Specifically, our analysis focused on the outcomes of patients with and without a CC.

## Methods

### Literature search strategy

We systematically searched five electronic databases, including PubMed, Cochrane Library, Medline, Web of Science, and EMBASE using the following combination of MeSH (Medical Subject Heading) terms and free text words: “cervical collar”, “anterior cervical discectomy and fusion” and “ACDF”. The search date was from when the databases were created to 1 October 2023. We did not restrict searches based on language or publication year. To prevent certain studies from being missed, we manually searched the bibliographies of RCTs, meta-analyses, and systematic reviews.

### Selection of studies

The process for study inclusion and exclusion was twofold. Studies were first selected based on the title and abstract, and if a decision could not be made from the summary, the full text was retrieved. When there was a disagreement between the two groups, the selection committee held discussions until a consensus was reached.

### Inclusion and exclusion criteria

The study inclusion criteria are as follows: (1). RCTs or comparative studies (2). Comparative studies on the efficacy of wearing a cervical collar after ACDF. (3). Studies comparing at least one of the following outcomes: neck disability index (NDI) scores, short form 36 (SF-36) questionnaire results, cervical range of motion (ROM) or fusion rate. The study exclusion criteria are as follows: (1). Editorials, letters, reviews, case reports, and cadaver or animal experiments. (2). Studies with patients diagnosed with scoliosis, infection or a tumour (3). Studies that did not meet the study inclusion criteria; and (4). Studies with data of the comparison outcomes that could not be extracted.

### Data extraction

Two reviewers used standardized data extraction tables. The extracted data included authors, publication date, title, country, study design, follow-up duration, number of patients, mean age of patients, type of operation, and comparison outcomes. The comparison of outcomes included the NDI score, SF-36 score, ROM, and fusion rate. All the data were extracted from article texts, tables, and figures. The research author was contacted for missing data or further information. Two reviewers independently extracted the data; differences were resolved through discussion, and a consensus was reached by a third party. The data extraction outcomes are shown in Table 1.

**Table 1:** Characteristics of included studies

Author (year)	Country	Study type	Number of Samples	Gender (male)	Average age	Follow-UP (months)	Surgical level	Outcomes
			CC/N-CC	CC /N-CC	CC /N-CC	CC /N-CC		
Campbell (2008)^11^	USA	RCT	149/108	65/53	44.3/43.3	24/24	1	1,2,3,4
Scerrati (2019)^12^	Italy	Cohort	36/36	17/18	48/48	12/12	1–2	1,4
Abbott (2013)^13^	Sweden	Cohort	17/16	9/11	53.4/47.3	24/24	–	1,2,3,4
Overley (2020)^14^	USA	RCT	22/22	12/12	55.2/50.1	12/12	1–2	1,4

*Outcomes*:1. Neck Disability Index, 2. Short Form 36, 3. cervical range of motion, 4. fusion rate

ZP: cervical collar ACP: no cervical collar RCT: Randomized controlled trial

### Data analysis

**Fig. 1:**
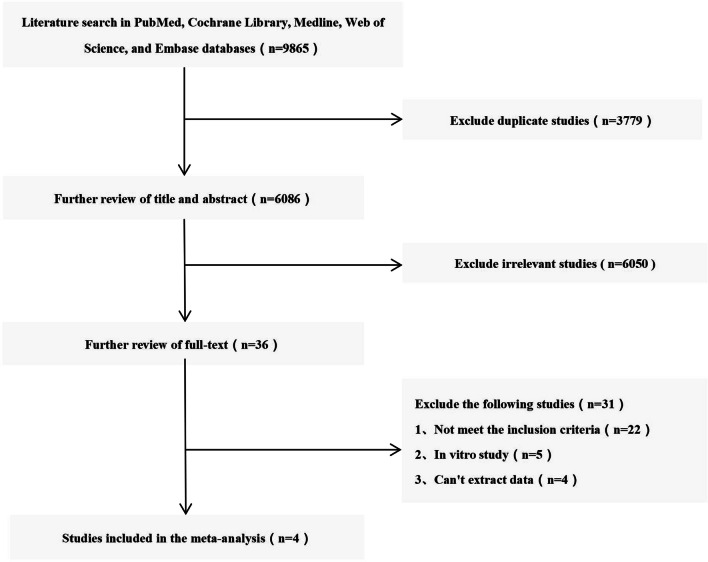
Flow diagram of study selection

We used Review Manager Version 5.4 (Copenhagen: The Nordic Cochrane Centre, The Cochrane Collaboration) to analyse the data for all outcomes and compare the CC group with the N-CC group. For continuous outcomes, such as NDI score, ROM and SF-36 score, the means and standard deviations were pooled to calculate the weighted mean difference (WMD) and 95% confidence intervals (CIs). Risk ratios (RRs) and 95% CIs were used to evaluate dichotomous outcomes, such as the fusion rate. We used *I*^2^ to quantify heterogeneity. If *I*^2^ > 50%, the heterogeneity was considered significant, and the unstandardized mean difference was estimated using a random effects model. Otherwise, a fixed-effects model was applied (Fig. 1).

### Quality assessment

**Fig. 2:**
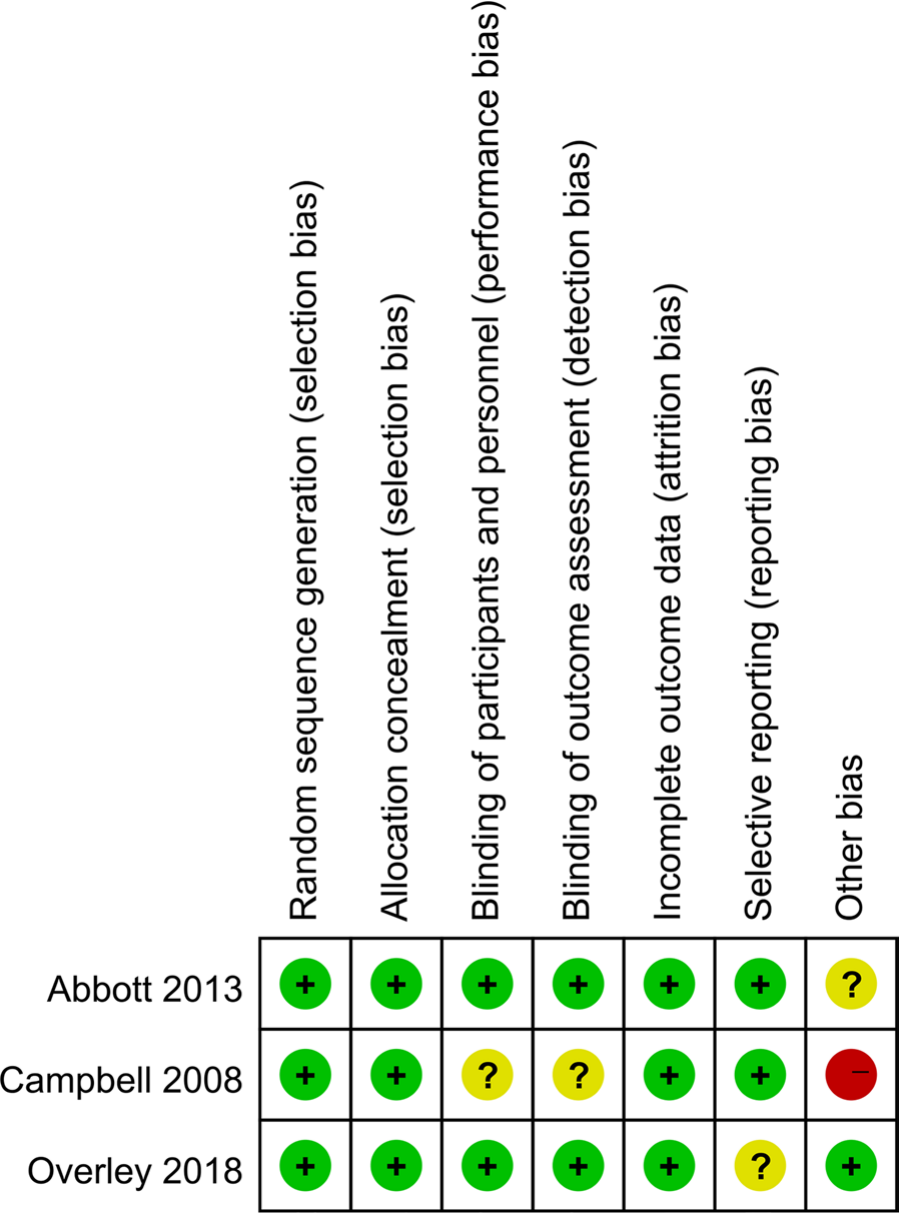
The methodological quality of the randomized controlled trials

For nonrandomized controlled trials (N-RCTs), the modified Newcastle Ottawa Scale (NOS) was used to assess the risk of bias [9]. Three domains in the NOS were evaluated, including selection, comparability, and exposure, totalling 9 points (Table 2). For RCTs, the Cochrane Handbook for Systematic Reviews of Interventions was used [10], which included 7 domains: random sequence generation, allocation concealment, blinding of participants and personnel, blinding of outcome assessment, incomplete outcome data, selective outcome reporting, and other sources of bias (Fig. 2). Two reviewers independently conducted the quality assessment and discussed disagreements with a third party.

**Table 2:** Quality assessment of cohort studies according to the Newcastle Ottawa Scale (NOS)

Author	Selection	Comparability	Exposur	Total score
Scerrati.et al	3	2	3	8

## Results

### Literature search

There were 9865 studies identified from five electronic databases (Fig. 1). Of those, 3779 studies were duplicates, and 6086 studies were excluded after title and abstract screening. After careful full-text evaluation, 4 studies [11–14] were reviewed, and the data were extracted. The demographic and clinical characteristics of the 4 studies are described in Table 1. The clinical data of 224 patients who wore a CC were compared with those of 182 patients who did not wear a CC. The mean follow-up time was more than 12 months, and the mean age of the patients was 43–55 years. The NDI score and fusion rate were reported in 4 studies [11–14]. The SF-36 score and ROM were reported in 2 studies [11, 13].

### NDI

**Fig. 3:**
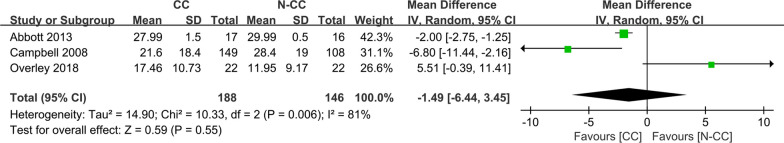
Meta-analysis of CC group versus N-CC group in NDI at 6 weeks after surgery

Three studies [11, 13, 14] with 188 and 146 patients compared the mean NDI score at 6 weeks after surgery between the CC and N-CC groups. The meta-analysis indicated no significant differences between the CC and N-CC groups (WMD, − 1.49; 95% CI − 6.44 to 3.45; *P* > 0.05). The heterogeneity test outcome (*I*^2^ = 88%) demonstrated significant heterogeneity (Fig. 3).

**Fig. 4:**
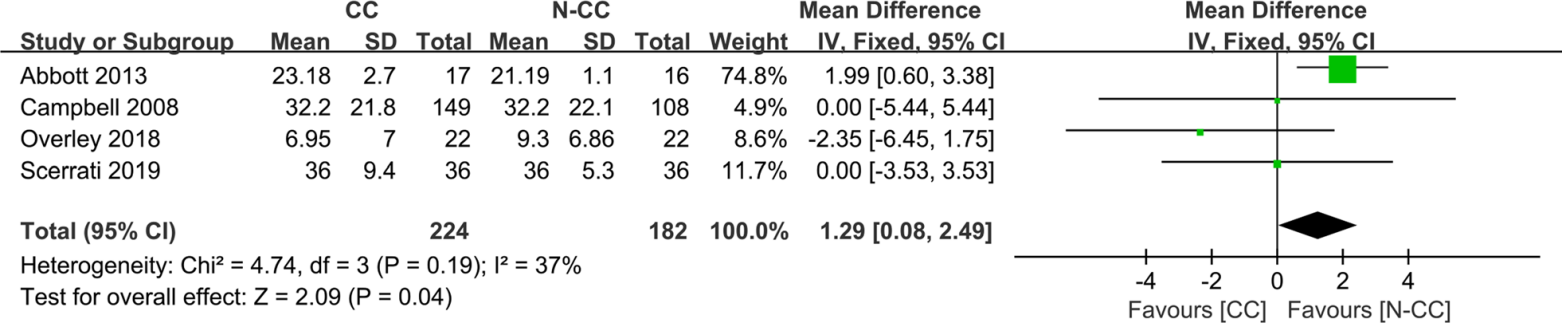
Meta-analysis of CC group versus N-CC group in NDI at the last follow-up

Four studies [11–14] with 224 and 182 patients, respectively, compared the mean NDI score at the last follow-up visit between the CC and N-CC groups. The pooled outcomes showed that the N-CC group had significantly lower NDI scores than the CC group had (WMD, 1.29; 95% CI, 0.08 to − 2.49; *P* < 0.05). The heterogeneity test outcome (*I*^2^ = 37%) and the fixed effects model were applied. The results showed that patients who did not wear a CC after ACDF surgery had significantly lower NDI scores than those who did wear a CC (Fig. 4).

### ROMs

**Fig. 5:**
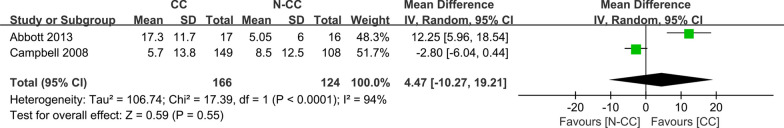
Meta-analysis of CC group versus N-CC group in ROM

Two studies [11, 13] with 166 and 124 patients compared the mean ROM between the CC and N-CC groups, respectively. The meta-analysis indicated no significant differences between the CC and N-CC groups (WMD, 4.47; 95% CI − 10.27 to 19.21; *P* > 0.05). The heterogeneity test outcome (*I*^2^ = 94%) demonstrated significant heterogeneity (Fig. 5).

### SF-36

**Fig. 6:**
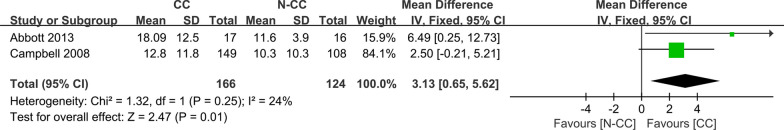
Meta-analysis of CC group versus N-CC group in SF-36

Two studies [11, 13] with 166 and 124 patients, respectively, compared the mean SF-36 score between the CC and N-CC groups. The pooled outcomes showed that the N-CC group had significantly lower SF-36 scores than the CC group had (WMD, 3.13; 95% CI 0.65 to 5.62; *P* < 0.05). The heterogeneity test outcome (*I*^2^ = 24%) and the fixed effects model were applied. The results showed that patients who did not wear a CC after ACDF had significantly lower SF-36 scores than those who did wear a CC (Fig. 6).

### Fusion rate

**Fig. 7:**
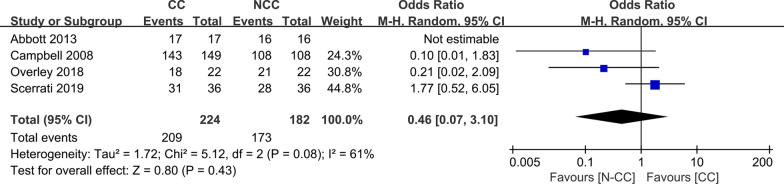
Meta-analysis of CC group versus N-CC group in fusion rate

Four studies [11–14] with 224 and 182 patients, respectively, compared the fusion rate between the CC and N-CC groups. The meta-analysis indicated no significant differences between the CC and N-CC groups (OR, 0.46; 95% CI 0.07 to 3.10; *P* > 0.05). The heterogeneity test outcome (*I*^2^ = 61%) demonstrated significant heterogeneity (Fig. 7).

## Discussion

ACDF is a widely accepted surgical method for the treatment of degenerative cervical spine diseases [15]. This procedure aims to decompress the spinal cord, improve the stability of the cervical spine, and have a positive impact on nerve roots [16]. The use of CCs has become a common practice in treating various clinical conditions such as postwhiplash injuries and in ACDF surgeries [17]. However, there is still a lack of scientific evidence to support this practice. Only a few comparative studies have examined the efficacy of CCs after ACDF for the treatment of cervical spondylosis.

In our meta-analysis, the information of 406 patients was extracted from 4 published studies, including three RCTs and one cohort, using the Cochrane Handbook for Systematic Reviews of Interventions and the NOS for quality assessment. The included studies were of high quality. Our study demonstrated that the N-CC group had significantly lower NDI and SF-36 scores than the CC group had at the last follow-up visit. However, meta-analysis of the mean 6-week NDI score, ROM, and fusion rate revealed no significant differences between the CC group and the N-CC group.

Compared to physical therapy protocols or usual care, wearing a CC has been found to be associated with worse clinical outcomes after whiplash injuries. Patients who wore a CC experienced more residual neck pain and restricted cervical ROM, particularly in the short-term follow-up period. According to Campbell et al. [11], patients who wore a CChad significantly higher NDI scores during the early postoperative period than patients who did not wear a CC. This finding suggests that the discomfort and disability associated with postoperative brace use may contribute to higher NDI scores. Our findings indicate that there were no significant differences in the 6-week mean NDI score between the CC group and the N-CC group, as observed from the meta-analysis. However, it is worth noting that the N-CC group had significantly lower NDI scores than the CC group had during the last follow-up visit. This may be attributed to the use of a CC, which restricts movement of the cervical spine. This restriction can lead to atrophy of the neck muscles, resulting in discomfort such as neck pain and soreness.

Limiting excessive motion can increase fusion rates and improve clinical outcomes. Surgeons initially applied cervical orthoses to achieve external immobilization. The principle of limiting excessive motion also motivated the introduction of anterior plating in ACDF. Our study demonstrated that there was no significant difference in the fusion rate between the CC group and the N-CC group. This finding aligns with the conclusion of Jagannathan et al.’s study [18], which had limited evidence, as it only investigated fusion rates in patients who did not wear a CC. Abbott et al. [13] conducted a study to examine the impact of CCs on two distinct patient groups. They observed that there were no qualitative differences in postoperative fusion rates between the group that wore a CC and the group that did not three months after surgery. Theoretically, wearing a CC can limit the movement of the cervical spine and promote cervical spine fusion. However, our research revealed no significant difference in the fusion effect between patients who wore a CC and those who did not wear a CC after ACDF surgery.

Like previous meta-analytic reports, this study is subject to several limitations. The studies included in this meta-analysis were not of high quality, as systematic reviews and meta-analyses should ideally rely on evidence from high-quality studies such as RCTs, well-structured prospective trials, and prospective cohort studies. Additionally, the sample size in this study was deemed insufficient.

## Conclusion

According to our analysis, wearing a CC after ACDF was found to be more beneficial in terms of the SF36 score. However, it is important to note that when considering the NDI score at the final follow-up visit, not wearing a CC was found to be more advantageous. There was no difference in the 6-week postoperative ROM, fusion rate, or NDI score between the CC group and the no CC group. It is important to note that further properly designed clinical trials are needed in the future to confirm these results and to investigate the long-term effects of wearing a CC after ACDF.

## Data Availability

All data generated or analysed during this study are included in this published article and its supplementary information files.

## Abbreviations

CDDD: Cervical degenerative disc disease
ACDF: Anterior cervical decompression and fusion
CC: Cervical collar
NDI: Neck disability index
SF-36: Short form 36
ROM: Cervical range of motion
RCTs: Randomized controlled trials
WMD: Weighted mean difference
CI: Confidence interval
RRs: Risk ratios
